# Regionale verschillen in het gebruik van urinekatheters in Nederland: een landelijke cohortstudie van 2012–2021

**DOI:** 10.1007/s13629-024-00458-w

**Published:** 2025-01-21

**Authors:** Felice E. E. van Veen, Jeroen R. Scheepe, Bertil F. M. Blok

**Affiliations:** Afdeling Urologie, Erasmus MC, Rotterdam, Nederland

**Keywords:** blaaskatheterisatie, intermitterende katheterisatie, onderactieve blaas, urineretentie, prevalentie, praktijkvariatie, Urinary catheterization, Clean intermittent catheterization, Underactive bladder, Urinary retention, Prevalence, Practice variation

## Abstract

**Inleiding:**

Schone intermitterende katheterisatie (CIC) wordt vaak verkozen boven verblijfskatheters (CAD) vanwege een lager risico op complicaties en verbeterde levenskwaliteit. Dit onderzoek richt zich op trends en regionale verschillen in het gebruik van CIC en CAD onder thuiswonenden in Nederland tussen 2012 en 2021.

**Methode:**

Er werden gegevens verzameld uit het Genees- en hulpmiddeleninformatieproject (GIP) en er werden regionale verschillen geëvalueerd met negatieve binomiale regressie (NBR).

**Resultaten:**

Het aantal CIC-gebruikers steeg met 27,3 % van 34.204 naar 43.528 en het aantal CAD-gebruikers steeg met 44,6 % van 41.619 tot 60.172. De grootste toenamen werden gezien bij mannelijke CIC-gebruikers > 65 jaar en mannelijke CAD-gebruikers > 85 jaar. NBR toonde significante regionale verschillen met een hoger CIC-gebruik in het noorden van Nederland en een variabel CAD-gebruik door het hele land.

**Conclusie:**

De resultaten wijzen op een groeiend aantal kathetergebruikers in Nederland. Daarnaast zijn er regionale verschillen, die mogelijk verklaard worden door verschillen in patiëntpopulaties, zorgverlenervoorkeuren en richtlijnnaleving.

## Abstract (en)

**Introduction:**

Clean intermittent catheterization (CIC) is often preferred over indwelling catheters (IDC) due to fewer complications and improved quality of life. This study investigated trends and regional differences in CIC and IDC use in the Netherlands between 2012 and 2021.

**Methods:**

Data were collected from the Drug and Medical Devices Information System and regional differences were assessed using negative binomial regression (NBR).

**Results:**

The number of CIC users increased by 27.3%, from 34,204 to 43,528 and the number of ID users by 44.6%, from 41,619 to 60,172. The most significant increases were among male CIC users > 65 years and IDC users > 85 years. NBR showed significant regional variations, with higher CIC use in the northern Netherlands and variable IDC use across the country.

**Conclusion:**

There is a growing number of catheter users in the Netherlands and there are regional differences, which may be explained by variations in patient populations, healthcare provider preferences, and adherence to guidelines.

## Inleiding

Blaaskatheterisatie wordt gebruikt bij de behandeling van urineretentie, het onvermogen om de blaas (volledig) te legen. Urineretentie kan zowel een neurogene als niet-neurogene oorzaak hebben. Een dwarslaesie, spina bifida, multipele sclerose en de ziekte van Parkinson zijn de meest voorkomende neurogene oorzaken [1]. Niet-neurogene oorzaken omvatten obstructie van de blaasuitgang (zoals BPH, urethrastrictuur, bekkenbodemdisfunctie), post partum urineretentie of postoperatieve urineretentie, of kunnen idiopathisch van aard zijn [2]. Het is van belang om onderscheid te maken tussen een neurogene en niet-neurogene oorzaak, aangezien de behandelstrategieën fundamenteel verschillen en de keuze voor katheterisatie kunnen beïnvloeden.

Schone intermitterende katheterisatie (CIC) wordt beschouwd als de voorkeursmethode voor blaasdrainage bij zowel neurogene als niet-neurogene patiënten [3, 4]. Vergeleken met verblijfskatheters (CAD) vermindert CIC het risico op kathetergerelateerde urineweginfecties, ongemak (blaaskrampen), blaasstenen en nierfunctieachteruitgang, terwijl de kwaliteit van leven wordt verbeterd door grotere onafhankelijkheid, mobiliteit en het behoud van seksuele activiteit [5, 6]. De keuze tussen CIC of CAD hangt af van diverse factoren, waaronder patiëntfactoren (onder andere onderliggende ziekte, gewicht, handfunctie, positie van de meatus, patiëntvoorkeur), de beschikbare zorg en de vergoeding van urinekatheters. De beslissing moet uiteindelijk op individueel niveau worden genomen, waarbij rekening wordt gehouden met de persoonlijke behoeften en omstandigheden van de patiënt.

De huidige besluitvorming met betrekking tot geassisteerde blaasdrainage is echter niet transparant of gestandaardiseerd. De keuze voor het soort katheter of het aanbieden van alternatieve behandelopties hangt waarschijnlijk af van de voorkeur van de zorgverlener, die meestal gebaseerd is op klinische ervaring en vertrouwdheid met specifieke katheterfabrikanten.

In Nederland is het aantal kathetergebruikers de afgelopen twee decennia aanzienlijk toegenomen [7, 8]. Het is echter niet bekend of er regionale verschillen zijn met betrekking tot het aantal kathetergebruikers in Nederland. Slechts één eerder onderzoek in Engeland heeft gekeken naar regionale verschillen in het aantal chronische CAD-gebruikers. De onderzoekers vonden een vergelijkbare prevalentie van kathetergebruikers in zowel het zuiden (0,146 %) als westen (0,141 %) van Engeland [9]. Onlangs is een artikel gepubliceerd in *Therapeutic Advances in Urology* over regionale verschillen in het extramuraal (niet-geïnstitutionaliseerd) gebruik van urinekatheters in Nederland tussen 2012 en 2021 [10]. In dit artikel worden de resultaten van dit landelijke cohortonderzoek samengevat.

## Methode

Voor dit retrospectieve, landelijke cohortonderzoek werden gegevens verzameld uit het Genees- en hulpmiddeleninformatieproject (GIP) van Zorginstituut Nederland. De GIP-databank bevat informatie over alle declaraties van extramurale (niet-geïnstitutionaliseerde) hulpmiddelenzorg en farmaceutische zorg voor middelen die zijn opgenomen in het basispakket van de Zorgverzekeringswet. Sinds de invoering van deze wet in 2006 bevat de GIP-databank alle declaraties van de hele Nederlandse bevolking. Declaraties van medische hulpmiddelen worden gecodeerd via ZI-nummers of Generieke Productcodes Hulpmiddelen (GPH) en vervolgens door zorgverzekeraars verstrekt aan de GIP-databank. De GIP-databank koppelt deze codes aan ISO9999-codes, die worden vertaald naar monitorclassificaties. Urinekatheters vallen onder de monitorcode A1535 katheters en hebben verschillende ISO-codes.

Voor dit onderzoek werden alle koppelingen tussen de ZI-nummers/GPH-codes en ISO-codes voor urinekatheters gecontroleerd, waarbij verkeerd geclassificeerde producten werden gecorrigeerd. Dit heeft geleid tot een definitieve indeling van de verschillende soorten katheters. Op basis hiervan werd het aantal kathetergebruikers tussen 2012 en 2021 vastgesteld en de verdeling van de gebruikers in die periode naar leeftijd en geslacht bepaald.

Kathetergebruikers konden worden ingedeeld naar provincie op basis van declaraties die gekoppeld waren aan 31 zorgkantoorregio’s van Nederland. Deze regio’s werden vervolgens onderverdeeld in de twaalf provincies volgens de Nomenclatuur van Territoriale Eenheden voor de statistiek (NUTS)-codes van Nederland [11]. Als een zorgkantoorregio uit gemeenten uit meer provincies bestond, werd de zorgkantoorregio toegewezen aan de provincie waartoe de meeste gemeenten behoorden.

De volgende gegevens over kathetergebruikers werden geëvalueerd:

1. het aantal thuiswonende CAD- en CIC-gebruikers in de totale Nederlandse bevolking van 2012 tot 2021;
2. de geslachts- en leeftijdsverdeling van thuiswonende CAD- en CIC-gebruikers in de totale Nederlandse bevolking in 2012;
3. het aantal thuiswonende CAD- en CIC-gebruikers per provincie van 2012 tot 2021;
4. de geslachts- en leeftijdsverdeling van thuiswonende CAD- en CIC-gebruikers per provincie in 2012 en 2021.

Om het verschil in het aantal kathetergebruikers tussen de provincies te analyseren, werd gecorrigeerd voor de leeftijds- en geslachtsverdeling van de bevolking per provincie per jaar, op basis van de bevolkingsgegevens van het Centraal Bureau voor de Statistiek (CBS) [12]. Op deze manier werd gecorrigeerd voor zowel de verschillen in demografische samenstelling tussen de provincies, als de veranderingen in de loop der jaren (zoals bevolkingsgroei en vergrijzing). Dit relatieve aantal kathetergebruikers werd uitgedrukt per 100.000 mensen in dezelfde leeftijds- en geslachtscategorie. Hiervoor werd de volgende formule gebruikt:

$$\begin{aligned}\text{Relatief aantal kathetergebruikers}=\\\left(\frac{\text{Aantal kathetergebruikers}}{\text{Totale bevolking}}\right)\times 100.000\end{aligned}$$

Daarbij werden ‘aantal kathetergebruikers’ en ‘totale bevolking’ per provincie, leeftijdscategorie, geslacht en jaar berekend.

Multivariabele negatieve binomiale regressiemodellen (NBR-modellen) werden gebruikt om verschillen in het aantal kathetergebruikers tussen de provincies aan te tonen, waarbij gecorrigeerd werd voor leeftijd en geslacht. In het NBR-model van CAD-gebruikers werd de leeftijdsgroep 0–45 jaar niet meegenomen, omdat de kleine aantallen in deze groep zouden leiden tot een onbetrouwbaar model.

Alle analyses werden uitgevoerd met SPSS Statistics versie 28.0 (IBM Corp, Armonk, NY, VS).

## Resultaten

### Kathetergebruikers: totale bevolking

Tussen 2012 en 2021 steeg het absolute aantal thuiswonende CAD-gebruikers met 44,6 % van 41.619 naar 60.172 gebruikers. Het aantal thuiswonende CIC-gebruikers nam met 27,3 % toe, van 34.204 naar 43.528 gebruikers. Figuur 1 toont de tijdtrend van thuiswonende kathetergebruikers in de totale bevolking van 2012 tot 2021. In 2019 en 2020 was er een afname in het absolute aantal CAD- en CIC-gebruikers, die werd veroorzaakt door ontbrekende declaratiegegevens van de op twee na grootste zorgverzekeraar van Nederland, de VGZ (Stichting Volksgezondheidszorg) Zorgverzekeringsgroep. Na het verwijderen van alle declaraties van de VGZ-groep uit de analyses, werd geen afname in het absolute aantal CAD- en CIC-gebruikers waargenomen, maar zoals verwacht een continue toename (fig. 1). Het relatieve aantal kathetergebruikers, gecorrigeerd voor bevolkingsgroei tussen 2012 en 2021, toonde ook een stijging bij zowel CAD-gebruikers als CIC-gebruikers. Het aantal CAD-gebruikers steeg met 38,3 % van 248 naar 343 gebruikers per 100.000 mensen. Voor CIC-gebruikers was de stijging 21,1 %, van 204 naar 247 per 100.000 mensen.

**Figuur 1:**
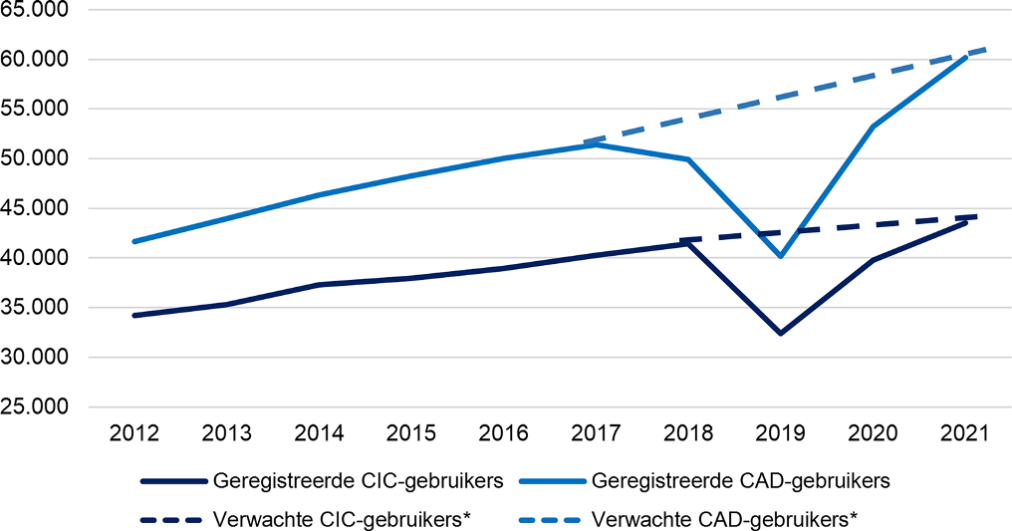
Aantal kathetergebruikers gecorrigeerd voor de totale bevolking van 2012 tot 2021. * In 2019 en 2020 werd het aantal kathetergebruikers onderschat door ontbrekende gegevens van zorgverzekeraars. Zonder deze ontbrekende gegevens zou er geen afname in kathetergebruikers zijn waargenomen

Figuur 2 toont de leeftijds- en geslachtsverdeling van CAD- en CIC-gebruikers in 2012 en 2021, en illustreert in één oogopslag het verhoogde risico op katheterisatie naarmate de leeftijd toeneemt. In de afgelopen tien jaar is het aantal mannelijke CIC-gebruikers in de leeftijdscategorieën 65–85 jaar en > 85 jaar aanzienlijk toegenomen, respectievelijk met 39,2 % (van 813 naar 1.131 gebruikers per 100.000 mannen) en 51,0 % (van 1.111 naar 1.677 gebruikers per 100.000 mannen). Het aantal mannelijke CIC-gebruikers tussen 45–64 jaar is toegenomen met 7,5 % (van 231 naar 248 gebruikers/100.000 mannen). In tegenstelling tot de trend bij mannen, is bij vrouwelijke CIC-gebruikers sprake van een kleine afname in alle leeftijdscategorieën. Het aantal vrouwelijke CIC-gebruikers tussen 0–45 jaar en 45–65 jaar is afgenomen met respectievelijk 10,4 % (van 75 naar 68 gebruikers per 100.000 vrouwen) en 6,7 % (van 216 tot 180 gebruikers per 100.000 vrouwen). Het aantal vrouwelijke CIC-gebruikers tussen 65–85 jaar en > 85 jaar bleef relatief stabiel met respectievelijk −3,4 % (van 410 naar 396 gebruikers per 100.000 vrouwen) en −2,4 % (van 466 naar 455 gebruikers per 100.000 vrouwen).

**Figuur 2:**
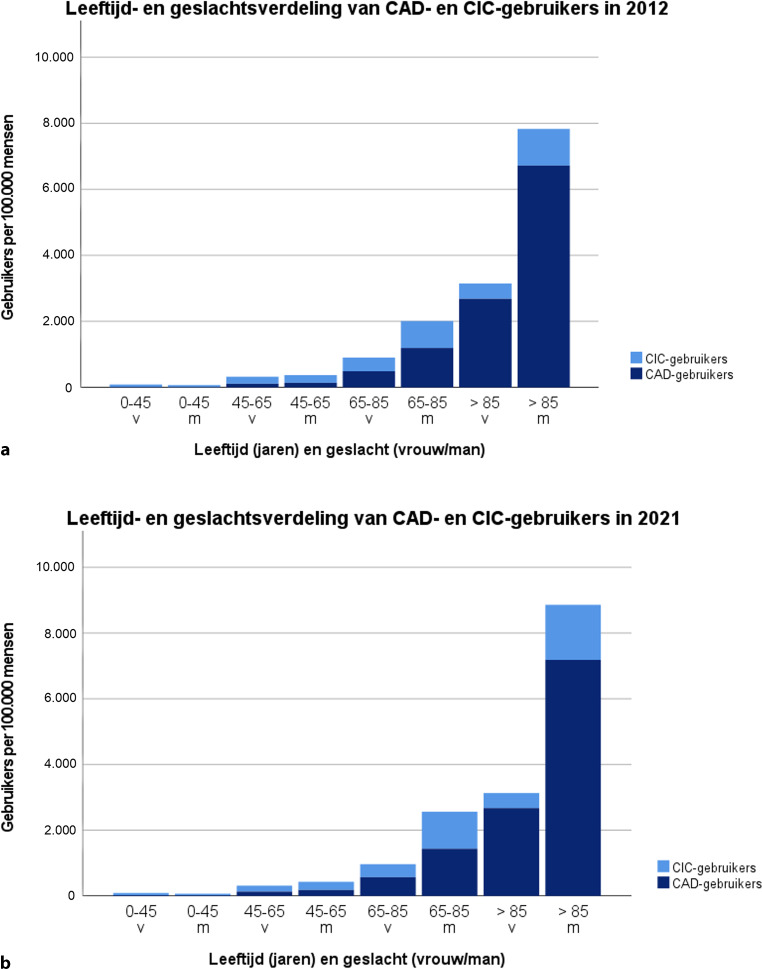
Leeftijds- en geslachtsverdeling van CIC- en CAD-gebruikers per 100.000 mensen in 2012 (**a**) en 2021 (**b**)

Voor CAD-gebruikers werd de grootste toename waargenomen in de leeftijdscategorie 0–45 jaar, met voor mannelijke gebruikers een stijging van 49,4 % (van 11 naar 17 gebruikers per 100.000 mannen) en voor vrouwelijke gebruikers 56,8 % (van 15 naar 23 gebruikers per 100.000 vrouwen). De grootste absolute toename werd echter gezien bij mannen ouder dan 85 jaar, namelijk van 6.721 naar 7.185 gebruikers per 100.000 mannen. In tegenstelling hiermee was er geen toename te zien bij vrouwelijke CAD-gebruikers van dezelfde leeftijdscategorie, waarin het aantal gebruikers stabiel bleef (van 2.685 naar 2.675 gebruikers per 100.000 vrouwen).

### Kathetergebruikers: per provincie

Figuur 3 laat zien hoe het aantal thuiswonende CIC- en CAD-gebruikers per provincie van 2012 tot 2021 is gestegen. De grootste stijging in CAD-gebruikers werd waargenomen in de provincies Brabant en Zeeland (het zuiden van Nederland), met respectievelijk een toename van 59 % en 50 %. Voor CIC-gebruikers was de grootste stijging te zien in de provincies Groningen en Drenthe (het noorden van Nederland), met een stijging van respectievelijk 39 % en 38 %. Gedurende deze periode nam ook de regionale variatie in het aantal CIC- en CAD-gebruikers toe. In 2012 varieerde het aantal CAD-gebruikers per provincie van 202 tot 304 gebruikers per 100.000 mensen, vergeleken met 239 tot 474 gebruikers per 100.000 mensen in 2021. Hetzelfde gold voor CIC-gebruikers, waarbij het aantal varieerde van 179 tot 277 gebruikers per 100.000 mensen in 2012, en 205 tot 382 gebruikers in 2021.

**Figuur 3:**
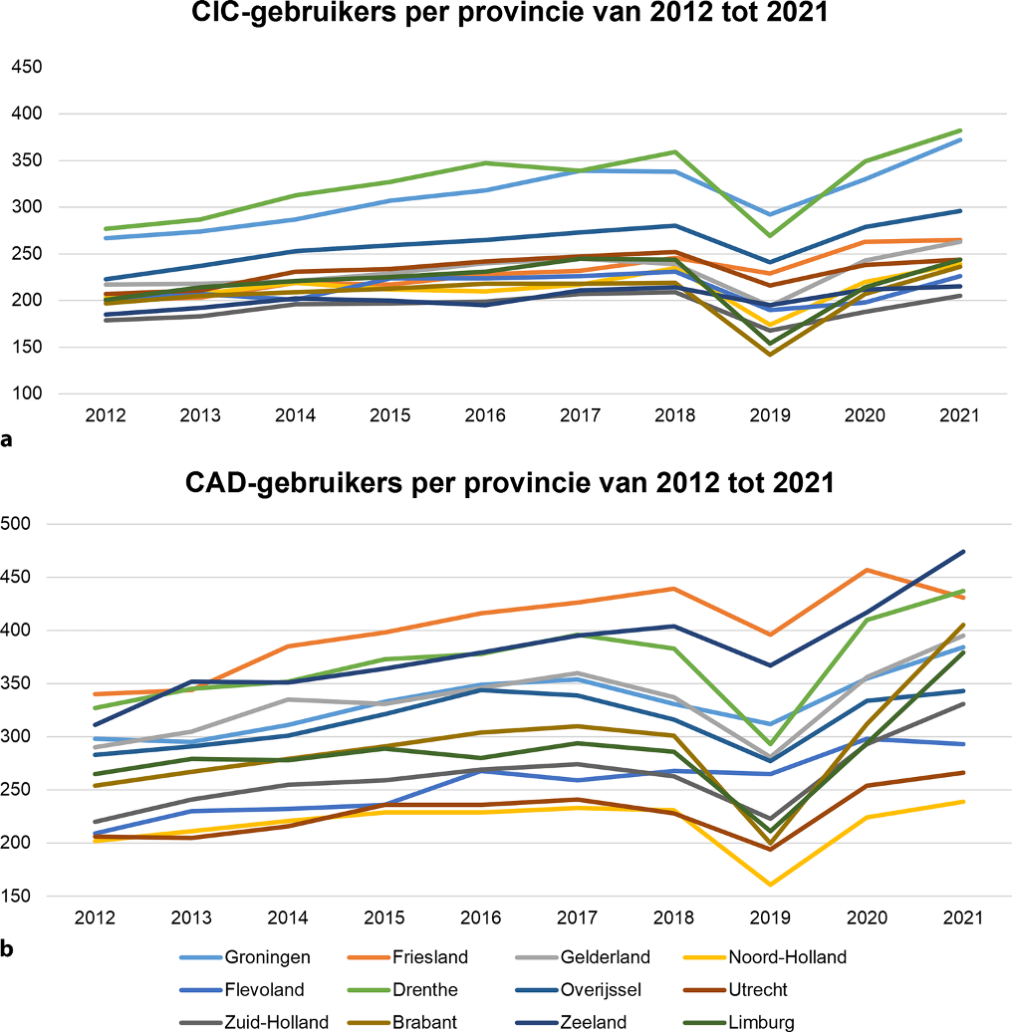
Het aantal CIC-gebruikers (**a**) en CAD-gebruikers (**b**) per 100.000 mensen per provincie van 2012 tot 2021

De geografische kaart in fig. 4 toont het verschil in aantal CIC- en CAD-gebruikers tussen de provincies in 2012 en 2021. De kaart toont een hogere prevalentie van CIC-gebruikers in het noorden van Nederland in 2021, terwijl een hoog aantal CAD-gebruikers geografisch meer verspreid was over het land.

**Figuur 4:**
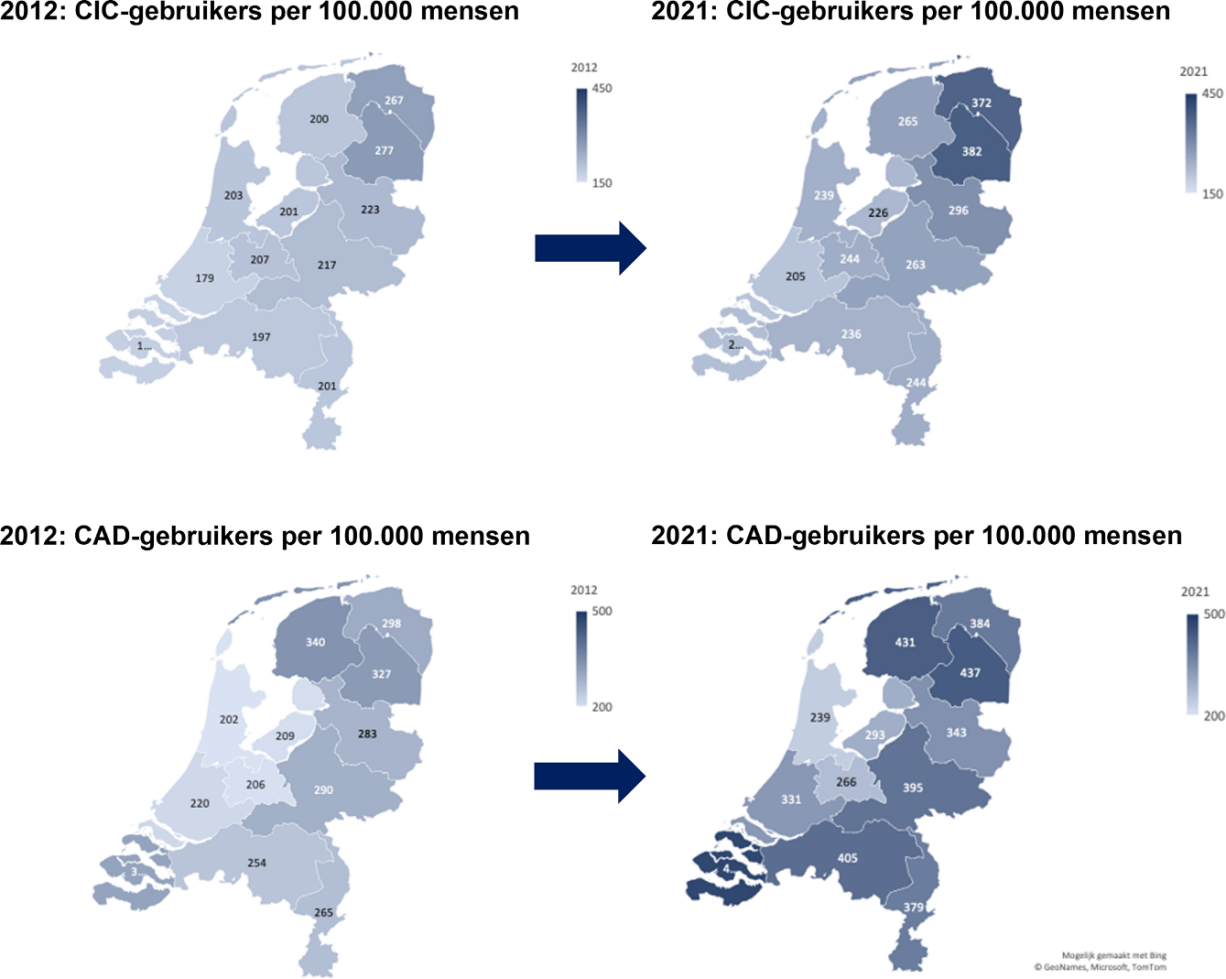
Verschillen in het aantal CIC- en CAD gebruikers tussen de provincies op de geografische kaart van Nederland in 2012 en 2021

De regionale verschillen in het aantal CAD-gebruikers lijken te worden bepaald door mannen en vrouwen > 85 jaar (fig. 5a), terwijl bij CIC-gebruikers vooral mannen in de leeftijdscategorieën 65–85 jaar en > 85 jaar een rol lijken te hebben gespeeld (fig. 5b).

**Figuur 5:**
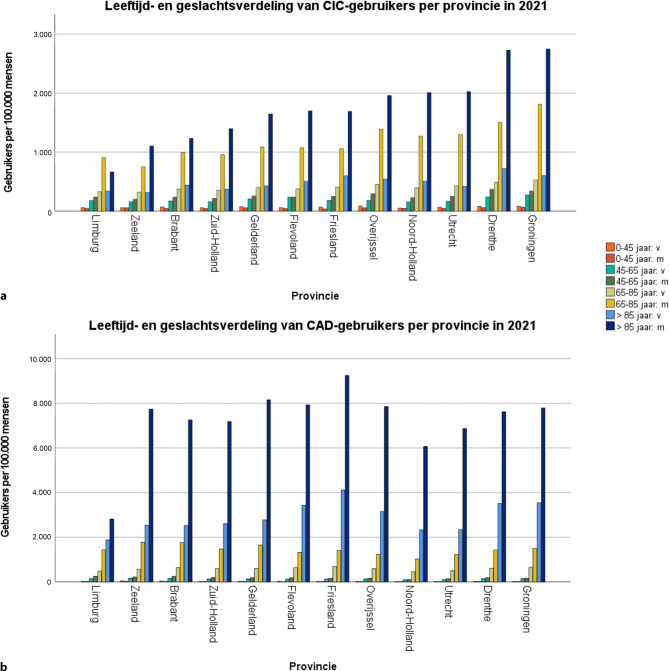
Leeftijd- en geslachtsverdeling van CIC-gebruikers (**a**) en CAD-gebruikers (**b**) per provincie in 2021

Multivariabele NBR-analyses toonden significante verschillen aan tussen de twaalf provincies voor CAD- en CIC-gebruikers (tab. 1 en 2). De incidentie van CIC-gebruikers was hoger in Drenthe en Groningen (het noorden van Nederland) in vergelijking met Zuid-Holland (IRR = 1,61, 95 %-BI 1,17–2,21, *p* = 0,003 en IRR = 1,67, 95 %-BI 1,22–2,29, *p* = 0,002, respectievelijk). Voor CAD-gebruikers was de incidentie significant hoger in zeven provincies verspreid over Nederland vergeleken met de provincie Noord-Holland (tab. 2). Daarnaast was de incidentie van zowel CIC- als CAD-gebruikers hoger bij mannen vergeleken met vrouwen, en nam de incidentie toe met de leeftijd.

**Tabel 1:** Resultaten van de negatieve binomiale regressieanalyse voor CIC-gebruikers in 2021

parameter	IRR (95 %-BI)	*p*-waarde
*provincie (referentie* *=* *Zuid-Holland)*		
Zeeland	0,93 (0,68–1,27)	0,641
Limburg	0,93 (0,68–1,28)	0,677
Brabant	1,08 (0,79–1,48)	0,634
Noord-Holland	1,16 (0,84–1,59)	0,369
Friesland	1,18 (0,86–1,62)	0,302
Flevoland	1,18 (0,86–1,62)	0,301
Gelderland	1,21 (0,88–1,67)	0,230
Utrecht	1,22 (0,89–1,67)	0,218
Overijssel	1,36 (0,99–1,87)	0,054
Drenthe	1,61 (1,17–2,21)	0,003
Groningen	1,67 (1,22–2,29)	0,002
*geslacht (referentie* *=* *vrouw)*		
man	1,80 (1,57–2,06)	< 0,001
*leeftijd (referentie* *=* *0–45 jaar)*		
45–65 jaar	3,35 (2,79–4,03)	< 0,001
65–85 jaar	10,37 (8,59–12,51)	< 0,001
> 85 jaar	13,97 (11,55–16,89)	< 0,001

**Tabel 2:** Resultaten van de negatieve binomiale regressieanalyse voor CAD-gebruikers in 2021

parameter	IRR (95 %-BI)	*p*-waarde
*provincie (referentie* *=* *Noord-Holland)*		
Utrecht	1,15 (0,91–1,45)	0,246
Limburg	1,17 (0,93–1,48)	0,176
Zuid-Holland	1,35 (1,07–1,70)	0,012
Overijssel	1,35 (1,07–1,71)	0,011
Gelderland	1,43 (1,13–1,80)	0,003
Flevoland	1,38 (1,10–1,74)	0,006
Groningen	1,47 (1,17–1,86)	0,001
Drenthe	1,50 (1,16–1,84)	0,001
Zeeland	1,50 (1,19–1,89)	< 0,001
Brabant	1,50 (1,19–1,89)	< 0,001
Friesland	1,50 (1,19–1,90)	< 0,001
*geslacht (referentie* *=* *vrouw)*		
man	2,03 (1,84–2,23)	< 0,001
*leeftijd (referentie* *=* *45–65 jaar)*		
65–85 jaar	5,72 (5,09–6,43)	< 0,001
> 85 jaar	28,43 (25,28–31,98)	< 0,001

## Discussie

Urinekatheters worden veel gebruikt bij de behandeling van een urineretentie. Dit onderzoek richtte zich op de trends en regionale verschillen in het extramurale (niet-geïnstitutionaliseerde) gebruik van urinekatheters in Nederland tussen 2012 en 2021. Onze resultaten laten zien dat het aantal CIC- en CAD-gebruikers de afgelopen jaren is blijven toenemen. Dit komt overeen met twee eerdere onderzoeken waarin het gebruik van urinekatheters werd geëvalueerd met een overlappend cohort van 1997–2018 [7, 8]. Deze vonden dat het aantal CIC-gebruikers verdrievoudigde en het aantal CAD-gebruikers verdubbelde over twee decennia. Daarnaast zagen ze ook een toename in het gebruik van CIC en CAD in de jongere leeftijdscategorieën, wat mogelijk te wijten is aan onder andere een exponentiële bevolkingsgroei.

In ons onderzoek werd alleen een toename gezien bij mannelijke CAD-gebruikers > 85 jaar en mannelijke CIC-gebruikers > 65 jaar. Deze toename bleef zichtbaar, zelfs na correctie voor vergrijzing, wat wijst op een bijdrage van zowel demografische factoren als urologische oorzaken. Daarnaast bleef verrassend genoeg het aantal vrouwelijke kathetergebruikers de afgelopen jaren stabiel. Een verschil tussen mannelijke en vrouwelijke kathetergebruikers werd ook gevonden in een eerder onderzoek naar de prevalentie van urinekatheters in Engeland [13]. Daar werd een twee keer hogere prevalentie van mannelijke kathetergebruikers in de totale bevolking gevonden. Dit contrasteert met andere veelgebruikte medische hulpmiddelen door oudere mensen, zoals hoortoestellen, waar geen verschil tussen de geslachten werd gezien [14]. Het lijkt daarom aannemelijk dat de toename van mannelijke kathetergebruikers verband houdt met specifieke urologische factoren.

Mannen hebben in vergelijking met vrouwen een langere urethra en kans op leeftijdgebonden prostaataandoeningen, zoals benigne prostaathyperplasie (BPH) en prostaatkanker. Door de vergrijzing neemt het aantal diagnoses van deze aandoeningen toe [15, 16]. In Nederland steeg het aantal prostaatkankerpatiënten tussen 2012 en 2022 van 104.000 naar 124.000, van wie 78 % 60 jaar of ouder was [16]. Deze stijging in prostaataandoeningen en eventuele behandelingen kan hebben bijgedragen aan het toenemende gebruik van urinekatheters bij oudere mannen.

De coronapandemie (COVID-19) had ingrijpende gevolgen voor onze gezondheidszorg en maatschappij in 2020 en 2021. Men zou kunnen veronderstellen dat COVID-19 impact heeft gehad op het extramurale (niet-geïnstitutionaliseerde) kathetergebruik door uitgestelde en aangepaste zorg. Desondanks hebben we tijdens deze periode geen veranderingen in het kathetergebruik waargenomen. Van Deukeren et al. toonden aan dat de impact van de eerste COVID-19-golf op mannen met de novo prostaatkanker beperkt was en dat het aantal radicale prostatectomieën in 2020 vergelijkbaar bleef met dat van de voorgaande jaren [17]. Alleen operaties voor goedaardige aandoeningen werden uitgesteld, wat resulteerde in een afname van het aantal transurethrale prostaatresecties met respectievelijk 1.500 en 2.500 procedures in 2020 en 2021 [18]. Deze afname had echter geen invloed op het totale kathetergebruik in de loop der tijd.

Onze resultaten toonden regionale verschillen in het gebruik van urinekatheters in Nederland: het gebruik van CIC was hoger in het noorden van Nederland en het gebruik van CAD varieerde tussen verschillende provincies. Deze bevindingen zijn opmerkelijk, omdat we hadden verwacht dat er in een relatief klein land met een uniforme urologieopleiding geen regionale verschillen zouden zijn. Een eerder onderzoek in Engeland toonde ook geen regionale verschillen in het aantal chronische CAD-gebruikers [9]. Verschillende factoren kunnen echter mogelijk de waargenomen variaties verklaren.

Ten eerste kunnen de variaties worden verklaard door verschillen in voorkeuren van zorgverleners en hun naleving van professionele richtlijnen. Volgens de richtlijnen van de European Association of Urology (EAU) en de American Urological Association (AUA) wordt CIC beschouwd als de voorkeursmethode voor blaasdrainage bij zowel neurogene als niet-neurogene patiënten [3, 4]. Aangezien CIC meer werd toegepast in het noorden van Nederland, is het mogelijk dat de zorgverleners daar de richtlijnen meer naleven. Een eerder onderzoek toonde aan dat slechts 53 % van de Nederlandse urologen de richtlijnen voor neurogene blaasstoornissen in de praktijk toepaste [19]. Bovendien wordt CIC in Nederland uitsluitend door urologen en revalidatieartsen voorgeschreven, terwijl CAD ook door andere zorgverleners kan worden voorgeschreven. Dit kan hebben bijdragen aan de grotere regionale verschillen bij CAD-gebruikers.

Ten tweede kunnen deze regionale verschillen worden verklaard door variaties in patiëntenpopulaties, zoals verschillen in onderliggende ziekten (neurogeen versus niet-neurogeen), demografische kenmerken en culturele achtergronden. Demografische factoren, zoals de sociaaleconomische status, kunnen per regio verschillen en invloed hebben op het kathetergebruik. Patiënten met een lage sociaaleconomische status kunnen bijvoorbeeld de voorkeur geven aan een CAD wanneer incontinentiemateriaal niet volledig wordt vergoed. Ook culturele overtuigingen en gewoonten kunnen een rol spelen bij de keuze tussen CIC of CAD [20]. In sommige delen van Noord-Nederland wordt mogelijk vaker gekozen voor CIC, wat zou kunnen samenhangen met een culturele nadruk op zelfstandigheid en pragmatisme. Dit zou ertoe kunnen leiden dat mensen de voorkeur geven aan een oplossing die hen in staat stelt om zelf meer controle te houden over hun zorg. Daarnaast kan de beschikbaarheid van zorgverleners of de bereidheid om hulp te accepteren ook van invloed zijn op de keuze.

Het inzichtelijk maken van regionale verschillen is essentieel voor beleidsmakers en zorgverleners, zodat ze gerichte interventies kunnen ontwikkelen en implementeren. Het identificeren van regio’s met een lager CIC-gebruik kan helpen bij het prioriteren van onderwijsinitiatieven en het bevorderen van evidence-based practice. Daarnaast zouden toekomstige interventies zich kunnen richten op het verbeteren en standaardiseren van het besluitvormingsproces voor blaaskatheterisatie, bijvoorbeeld door het introduceren van een digitale katheterkeuzehulp.

De belangrijkste beperking van dit onderzoek is het ontbreken van gegevens over het gebruik van urinekatheters in ziekenhuizen en zorgcentra. Hierdoor zal het daadwerkelijke gebruik van katheters, in het bijzonder verblijfskatheters, aanzienlijk hoger zijn in Nederland. Een andere beperking is dat de GIP-databank alleen informatie bevat over het aantal kathetervoorschriften. Gegevens over de duur (bijvoorbeeld chronisch of tijdelijk gebruik) en de indicatie van het kathetergebruik ontbreken, terwijl deze gegevens de regionale verschillen zouden kunnen helpen verklaren.

Daarnaast zijn de resultaten beperkt generaliseerbaar, aangezien de meeste katheters in Nederland door zorgverzekeraars vergoed worden. Dit geldt niet voor alle landen, zoals lage-inkomenslanden, waar sociaaleconomische factoren de keuze van het kathetertype kunnen beïnvloeden.

## Conclusie

Dit is het eerste onderzoek naar regionale verschillen in het extramurale (niet-geïnstitutionaliseerde) gebruik van urinekatheters in Nederland. In de afgelopen tien jaar is het aantal extramurale (niet-geïnstitutionaliseerde) kathetergebruikers in Nederland toegenomen, voornamelijk bij oudere mannen. Dit komt waarschijnlijk door hun anatomie en vatbaarheid voor leeftijdgebonden prostaatziekten, zoals BPH en prostaatkanker. Daarnaast zijn er regionale verschillen in het gebruik van katheters, met een hoger CIC-gebruik in het noorden van Nederland en een variërend CAD-gebruik tussen verschillende provincies. Deze variatie is mogelijk toe te schrijven aan verschillen in patiëntpopulaties en klinische praktijken, waaronder voorkeuren van zorgverleners en hun naleving van richtlijnen. Onze bevindingen onderstrepen de noodzaak van interventies gericht op het verbeteren en standaardiseren van de zorg en besluitvorming rond blaaskatheterisatie.
